# Ischemic cardio-cerebrovascular disease and all-cause mortality in Chinese elderly patients: a propensity-score matching study

**DOI:** 10.1186/s40001-024-01929-x

**Published:** 2024-06-15

**Authors:** Qian Yang, Shasha Sun, Long-Biao Cui, Shan Gao, Zhenghui Gu, Zhiyi Fang, Yingjie Zhang, Sijia Chen, Naiyuan Sun, Yabin Wang, Feng Cao

**Affiliations:** 1 Medical School of Chinese PLA, Beijing, 100039 China; 2 Department of Cardiology, The Second Medical Center & National Clinical Research, Center for Geriatric Diseases, Chinese PLA General Hospital, Beijing, 100853 China; 3 The Fifth Department of Cadre Health Care, The Second Medical Center & National Clinical Research Center for Geriatric Diseases, Chinese PLA General Hospital, Beijing, 100853 China; 4 School of Medicine, Nankai University, 94 Weijin Road, Tianjin, 30071 China

**Keywords:** Poly-vascular disease, Ischemic cardio-cerebrovascular disease, Mortality risk, Cohort study

## Abstract

**Background:**

Ischemic cardio-cerebrovascular disease is the leading cause of mortality worldwide. However, studies focusing on elderly and very elderly patients are scarce. Hence, our study aimed to characterize and investigate the long-term prognostic implications of ischemic cardio-cerebrovascular diseases in elderly Chinese patients.

**Methods:**

This retrospective cohort study included 1026 patients aged ≥ 65 years who were categorized into the mono ischemic cardio-cerebrovascular disease (MICCD) (either coronary artery disease or ischemic stroke/transient ischemic attack) (*n* = 912) and the comorbidity of ischemic cardio-cerebrovascular disease (CICCD) (diagnosed with both coronary artery disease and ischemic stroke/transient ischemic attack at admission) (*n* = 114). The primary outcome was all-cause death. The mortality risk was evaluated using the Cox proportional hazards risk model with multiple adjustments by conventional and propensity-score-based approaches.

**Results:**

Of the 2494 consecutive elderly patients admitted to the hospital, 1026 (median age 83 years [interquartile range]: 76.5–86.4; 94.4% men) met the inclusion criteria. Patients with CICCD consisted mostly of very elderly (79.2% vs. 66.1%, *P* < 0.001) individuals with a higher burden of comorbidities. Over a median follow-up of 10.4 years, 398 (38.8%) all-cause deaths were identified. Compared with the MICCD group, the CICCD group exhibited a higher adjusted hazard ratio (HR) (95% confidential interval, CI) of 1.71 (1.32–2.39) for long-term mortality after adjusting for potential confounders. The sensitivity analysis results remained robust. After inverse probability of treatment weighting (IPTW) modeling, the CICCD group displayed an even worse mortality risk (IPTW-adjusted HR: 2.07; 95% CI 1.47–2.90). In addition, anemia (adjusted HR: 1.48; 95% CI 1.16–1.89) and malnutrition (adjusted HR: 1.43; 95% CI 1.15–1.78) are also independent risk factors for all-cause mortality among elderly and very elderly patients.

**Conclusions:**

Our results thus suggest that elderly patients with ischemic cardio-cerebrovascular disease and anemia or malnutrition may have higher mortality, which may be predicted upon admission. These findings, however, warrant further investigation.

**Graphical Abstract:**

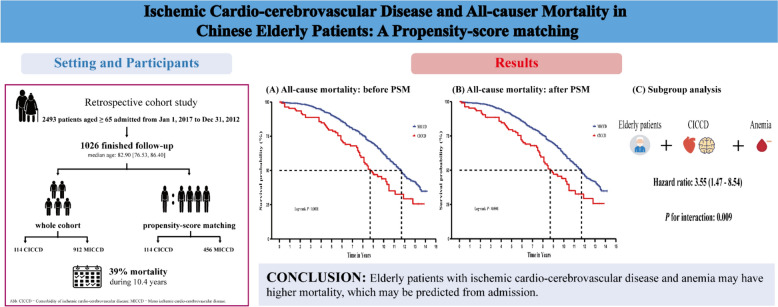

**Supplementary Information:**

The online version contains supplementary material available at 10.1186/s40001-024-01929-x.

## Introduction

Ischemic cardio-cerebrovascular disease, one of the leading causes of death worldwide, is a broad spectrum of serious health conditions including cardiovascular disease, hypertension, stroke, transient ischemic attack (TIA), and atherosclerosis [1, 2]. Their morbidity and mortality rates eclipse those of neoplastic conditions, thereby ascending to the foremost rank [3]. Physiologically, the heart and brain are profoundly interconnected; the cerebral tissue fundamentally depends on cardiac-driven blood circulation to sustain its regular functions. In the past few decades, the pathogenesis and clinical features of cardiovascular diseases have been extensively investigated, and it is believed that cardiovascular and cerebrovascular diseases have a common pathological basis. An empirical study reported that approximately 10–45% of the patients with cardiac issues might experience a stroke, 78.1–90.2% of those with cerebrovascular anomalies have an abnormal electrocardiogram, and 12.7% may be complicated by cerebral infarctions [4].

The concept of the "cardio-cerebrovascular continuum" delineates a state characterized by an increased likelihood of fatal progression in the absence of interventions that disrupt this pathological cycle [5]. Contemporary advancements, particularly in the domains of potent antithrombotic and lipid-lowering treatments, have markedly altered the prognosis of these patients. Nevertheless, despite implementing secondary prevention measures in patients diagnosed with coronary artery disease or cerebrovascular disease, the criteria for systematic pan-vascular screening and the judicious administration of newly developed therapies remain contentious. This ongoing debate may lead to underestimating risk and, consequently, undertreatment [6].

The prevalence of atherosclerotic involvement in extracardiac vascular beds is prominent among elderly patients and is an indicator of systemic atherosclerosis, thereby posing an increased risk for subsequent ischemic events [7]. Systemic atherosclerotic burden, such as myocardial infarction, coronary artery disease, and peripheral arterial disease, has consistently been reported as an independent risk factor for adverse cardiovascular events and short-term outcomes across various populations. These deleterious effects are largely consistent regardless of age and other vascular risk factors [8–11]. With the increase in life expectancy, ischemic cardio-cerebrovascular diseases have become significant conditions that severely impact the quality of life and survival of elderly and very elderly patients [12]. Consequently, the management of these patients has become a major concern. In 2019, the life expectancy of the Chinese population reached 77.3 years, and it is projected to increase to 81.3 years by 2035 [13]. During the urbanization and social transition processes, the effects of ischemic cardio-cerebrovascular diseases on the life expectancy of the oldest-old (≥ 80 years) patients remain unclear. Therefore, this study aims to evaluate the long-term prognosis of elderly patients with ischemic cardio-cerebrovascular diseases to improve their management in tertiary hospitals.

## Methods

### Study design and population

This study was conducted at the Second Medical Center of the Chinese PLA General Hospital. A total of 2493 consecutive elderly patients (over 65 years of age) who were admitted from January 1, 2007, to December 31, 2012, were enrolled in this retrospective aging-related cohort study. The inclusion criteria were (1) age ≥ 65 years; (2) a diagnosis of one of the ischemic cardio-cerebrovascular diseases, including coronary artery disease, ischemic stroke/TIA; (3) a diagnosis of both of these diseases were defined as the comorbidity of ischemic cardio-cerebrovascular disease (CICCD). The exclusion criteria were (1) no diagnosis of ischemic cardio-cerebrovascular disease; (2) known severe renal impairment (eGFR < 30 mL/min/1.73m^2^); (3) moderate to severe heart failure (NYHA III–IV); (4) significant hepatic disease (eg, ALT > 3 × upper limit of normal, cirrhosis, ascites); (5) malignancy (except non-metastatic, non-melanoma skin cancers, cervical in-situ carcinoma, breast ductal carcinoma in situ, or stage 1 prostate carcinoma); (6) no data on total cholesterol (TC), triglycerides (TG), high-density lipoprotein (HDL), and/or low-density lipoprotein (LDL) measurements; (7) no medication records; (8) potential cardioembolic stroke classified by the CISS classification [14]; and (9) no censoring information. The study protocol complied with the tenets of the Declaration of Helsinki and was approved by the ethics committee of the Chinese People’s Liberation Army General Hospital (S2021-326-02). Due to this study's retrospective nature, the ethics committee waived the need for written informed consent of the included patients. Meanwhile, we de-identified and anonymized patient records/information before analysis.

### Definition of ischemic cardio-cerebrovascular diseases

In this study, ischemic cardio-cerebrovascular disease was used as a general term for (1) coronary artery disease; and (2) cerebrovascular disease. Coronary artery disease was defined based on (1) physician diagnosis; (2) history of myocardial infarction, coronary artery bypass grafting, or percutaneous coronary intervention; and (3) treatment with statins. Cerebrovascular disease was defined as ischemic stroke or TIA [15]. Comorbidity of ischemic cardio-cerebrovascular disease was defined as a diagnosis with both coronary artery disease and cerebrovascular disease. A panel of three physicians reviewed patient medical records to confirm the diagnosis of the abovementioned diseases.

The main parameter in this study was the number of ischemic cardio-cerebrovascular diseases a patient was diagnosed with, based on which they were categorized into the following groups: (1) mono ischemic cardio-cerebrovascular disease; and (2) comorbidity of ischemic cardio-cerebrovascular disease.

### Endpoints and follow-up

For follow-up, the hospital’s electronic medical services were used. Considering the cause of death was not consistently available in our clinical cohort, and therefore we included all-cause and not cardiovascular mortality. Follow-up commenced on the day of admission and ended at the time of experiencing death or censoring events. Patients were censored for the event of interest, death, loss to follow-up, or December 31, 2020, whichever occurred first. Data on mortality, censoring events, and loss to follow-up were extracted from the hospital’s electronic medical record database. Lost to follow-up was determined based on the last date of active use of hospital electronic medical services (inpatient, outpatient, or laboratory).

### Data collection and definition

Information on baseline patient demographic characteristics, anthropometric measurements, smoking status, laboratory values, comorbid conditions, and medication use were extracted from the hospital’s electronic medical record database. Demographic characteristics included age and sex. Anthropometric measurements included height and weight. Body mass index (BMI) was calculated as weight (kg) divided by the square of height (m). BMI was categorized into four levels [16]: underweight (< 18.5 kg/m^2^), normal (18.5–23.9 kg/m^2^), overweight (24–27.9 kg/m^2^) and obese (≥ 28 kg/m^2^). Smoking status was classified into three categories: never, past, and current smoking. Laboratory values comprised measurements of hemoglobin, C-reactive protein (CRP), serum creatinine, urine acid, albumin, B-type natriuretic peptide (BNP), TC, TG, HDL, LDL, and glycated hemoglobin (HbA1c). The estimated glomerular filtration rate (eGFR) was calculated using the CKD Epidemiology Collaboration formula [17]. For laboratory values that were repeated, the mean value was included in analyses. Medications included aspirin, statins, ADP receptor inhibitors, anticoagulants, renin–angiotensin–aldosterone system inhibitors (RAASi), beta-blockers, and calcium channel blockers (CCBs).

The burden of other comorbidities was assessed by the age-adjusted Charlson community index (CCI) [18], an easily replicable approach, which was among the first indexes to determine disease severity and was widely used to assess physical functioning. The International Classification of Diseases, 10th Revision, was used to identify relevant diagnoses in the CCI disease inventory. Diabetes was identified by fasting blood glucose ≥ 7 mmol/L, random blood glucose ≥ 11.1 mmol/L, HbA1c ≥ 6.5%, or hypoglycemic medication use. Hypertension was defined as systolic blood pressure ≥ 140 mmHg, diastolic blood pressure ≥ 90 mmHg, or the current use of antihypertensive medications. CKD was defined as eGFR < 60 mL/min/1.73m^2^. Chronic pulmonary disease includes a history of chronic obstructive pulmonary disease or asthma.

### Statistical analysis

Patients’ baseline demographic and clinical characteristics were presented as mean ± standard deviation, median (interquartile range [IQR]), or percentages as appropriate for the total cohort and stratified by ischemic cardio-cerebrovascular disease numbers. Continuous variables were compared using a one-way analysis of variance or the Kruskal–Wallis test, as deemed appropriate. Categorical variables were compared using the chi-square test. Time-to-first event data were graphically presented using the Kaplan–Meier method. Cox proportional hazards regression was employed to calculate hazard ratios (HRs) with 95% CIs to examine the link between the comorbidity of ischemic cardio-cerebrovascular disease and all-cause mortality. The proportional hazard assumption was checked using Schoenfeld residuals. Before performing multivariable Cox proportional hazards regression analysis, the Mann–Whitney U test was used to ascertain the association between continuous variables in participants with the outcomes. Furthermore, Cox regression analysis was used to determine the association between the outcomes and each of the categorical variables. Variables for the multivariable Cox regression analysis were selected based on clinical knowledge or a significance probability of *P* < 0.2. To establish whether the continuous variables should be included as continuous or categorical variables in the model, restricted cubic splines were used to inspect nonlinear effects. The fully adjusted model included age, sex, BMI, smoking status, hemoglobin, CRP, albumin, BNP, TG, HDL, LDL, HbA1c, eGFR, history of hypertension or cancer, aspirin, ADP receptor inhibitors, beta-blockers, CCBs, and CCI-score. Missing category data on patients’ smoking status constituted 2.7% and were considered nonsmokers. Less than 5% of the baseline urine acid and HbA1c data were missing. Assumed to be missing at random, these data were imputed with multiple imputation of chained equations method using the R package “mice” (version 3.16).

To test the robustness and consistency of our findings, three sensitivity analyses were performed: (1) inverse probability of treatment weights (IPTW) was applied to balance confounding between two groups. The propensity score (PS) was determined by multivariable-adjusted logistic regression analysis using the variables listed in Table 1. IPTW was calculated by PS. Patients with CICCD and controls were matched 1:4 using the “greedy” nearest neighbor matching algorithm without replacement, with a caliper size of 0.05. The risk of mortality was tested by IPTW-based fully adjusted Cox regression model; (2) the fully adjusted model tested in male participants was used; and (3) to reduce the possibility of reverse causation in the long-term mortality analysis, patients who died within the 1st year of follow-up were excluded from the fully adjusted model.

**Table 1:** Patients’ characteristics before and after propensity score matching

Variables	Before PSM				After PSM			
	MICCD	CICCD	*P*-value^b^	SMD	MICCD	CICCD	*P*-value^b^	SMD
	N = 912^a^	N = 114^a^			N = 456^a^	N = 114^a^		
Age, year	82.65 (75.80, 86.32)	84.65 (81.03, 87.65)	** < 0.001**	0.404	84.70 (81.50, 87.70)	84.65 (81.03, 87.65)	0.711	0.066
Male, *n* (%)	862 (94.52)	106 (92.98)	0.650	0.063	424 (92.98)	106 (92.98)	1.000	< 0.001
BMI category, *n* (%)			0.131	0.221			0.929	0.068
Normal	402 (44.08)	53 (46.49)			212 (46.49)	53 (46.49)		
Underweight	25 (2.74)	7 (6.14)			22 (4.82)	7 (6.14)		
Overweight	436 (47.81)	51 (44.74)			212 (46.49)	51 (44.74)		
Obese	49 (5.37)	3 (2.63)			10 (2.19)	3 (2.63)		
Smoking, *n* (%)			0.259	0.171			1.000	0.024
Never	582 (63.82)	81 (71.05)			321 (70.39)	81 (71.05)		
Current	55 (6.03)	4 (3.51)			18 (3.95)	4 (3.51)		
Ever	275 (30.15)	29 (25.44)			117 (25.66)	29 (25.44)		
Hemoglobin, g/L	134.00 (124.00, 145.00)	136.00 (124.25, 145.00)	0.992	0.006	133.50 (124.00, 145.00)	136.00 (124.25, 145.00)	0.964	0.001
CRP, mg/dl	0.30 (0.13, 0.53)	0.31 (0.12, 0.73)	0.620	0.039	0.29 (0.13, 0.45)	0.31 (0.12, 0.73)	0.341	0.042
Creatinine, umol/L	83.00 (71.00, 98.00)	86.50 (69.00, 99.00)	0.924	0.104	85.00 (71.00, 98.00)	86.50 (69.00, 99.00)	0.955	0.009
Uric acid, umol/L	341.10 (284.90, 399.00)	331.10 (278.25, 374.75)	0.182	0.136	334.35 (275.50, 381.88)	331.10 (278.25, 374.75)	0.903	0.008
Albumin, g/L	41.70 (38.68, 44.60)	41.40 (38.47, 44.30)	0.540	0.056	41.60 (38.30, 44.42)	41.40 (38.47, 44.30)	0.971	< 0.001
BNP, pg/ml	126.60 (61.03, 286.55)	141.10 (83.27, 351.02)	0.078	0.110	135.30 (64.97, 346.72)	141.10 (83.27, 351.02)	0.417	0.035
Total cholesterol, mmol/L	4.06 (3.50, 4.71)	4.22 (3.81, 4.93)	**0.019**	0.226	4.24 (3.63, 4.97)	4.22 (3.81, 4.93)	0.753	0.021
Triglycerides, mmol/L	1.24 (0.91, 1.65)	1.18 (0.91, 1.64)	0.525	0.041	1.23 (0.90, 1.66)	1.18 (0.91, 1.64)	0.753	0.014
HDL, mmol/L	1.17 (0.96, 1.41)	1.25 (1.08, 1.47)	**0.023**	0.183	1.24 (1.01, 1.48)	1.25 (1.08, 1.47)	0.710	0.005
LDL, mmol/L	2.41 (1.96, 2.96)	2.54 (2.05, 3.13)	0.099	0.165	2.50 (2.01, 3.13)	2.54 (2.05, 3.13)	0.784	0.012
HbA1c, %	6.10 (5.80, 6.50)	6.00 (5.80, 6.60)	0.459	0.165	6.10 (5.80, 6.50)	6.00 (5.80, 6.60)	0.872	0.053
eGFR, ml/min per 1.73 m^2^	75.33 (60.62, 83.61)	68.28 (59.52, 83.50)	0.246	0.078	71.54 (59.68, 80.90)	68.28 (59.52, 83.50)	0.785	0.026
CCI score	3.00 (3.00, 4.00)	4.00 (3.00, 4.00)	** < 0.001**	0.321	4.00 (3.00, 5.00)	4.00 (3.00, 4.00)	0.821	0.014
Cancer, *n* (%)	59 (6.47)	1 (0.88)	**0.029**	0.301	2 (0.44)	1 (0.88)	1.000	0.054
Hypertension, *n* (%)	580 (63.60)	79 (69.30)	0.274	0.121	313 (68.64)	79 (69.30)	0.982	0.014
Atrial fibrillation, *n* (%)	51 (5.59)	6 (5.26)	1.000	0.015	25 (5.48)	6 (5.26)	1.000	0.010
Medication								
Aspirin, *n* (%)	429 (47.04)	65 (57.02)	0.056	0.201	263 (57.68)	65 (57.02)	0.983	0.013
ADP receptor inhibitor, *n* (%)	267 (29.28)	44 (38.60)	0.053	0.198	172 (37.72)	44 (38.60)	0.948	0.018
Statin, *n* (%)	463 (50.77)	74 (64.91)	**0.006**	0.289	288 (63.16)	74 (64.91)	0.811	0.037
Anticoagulants, *n* (%)	16 (1.75)	3 (2.63)	0.715	0.060	11 (2.41)	3 (2.63)	1.000	0.014
Beta blocker, *n* (%)	154 (16.89)	17 (14.91)	0.689	0.054	71 (15.57)	17 (14.91)	0.977	0.018
ACEI/ARB, *n* (%)	357 (39.14)	46 (40.35)	0.883	0.025	182 (42.11)	46 (40.35)	0.815	0.036
CCB*,* *n* (%)	454 (49.78)	68 (59.65)	0.059	0.199	269 (58.99)	68 (59.65)	0.983	0.013

^a^Median (IQR) or Frequency (%)

^b^Kruskal–Wallis rank sum test; Pearson’s Chi-squared test or Fisher’s exact test

PSM,  Propensity score matching; SMD, Standard mean difference; MICCD, Mono ischemic cardio-cerebrovascular disease; CICCD, Comorbidity of ischemic cardio-cerebrovascular disease; BMI, Body mass index; CRP, C-reactive protein; HDL, High density lipoprotein; LDL, Low density lipoprotein; eGFR, estimated glomerular filtration rate; CCI, Charlson Comorbidity Index; CCB, Calcium channel blocker

Post hoc subgroup analysis was performed to explore whether the potential association between comorbidity of ischemic cardio-cerebrovascular disease and outcomes was moderated by the following clinical characteristics: age (≥ 80 and 65–79 years) [16], BMI (≥ 24 and < 24), smoking, hemoglobin (≤ 120 and > 120 g/L in men, ≤ 110 and > 110 g/L in women) [19], CRP (≥ 0.3 and < 0.3 mg/dL) [20], albumin (< 40 and ≥ 40 g/L) [21], TG (≥ 1.2 and < 1.2 mmol/L) [22], LDL (≥ 1.8 and < 1.8 mmol/L), eGFR (< 60 and ≥ 60 mL/min per 1.73 m^2^), hypertension, aspirin, ADP receptor inhibitors, statins, and CCBs.

Statistical analyses were conducted using SPSS 25.0 (SPSS, Inc.) and R version 4.2.1 (The R Project for Statistical Computing). Two-sided *P* < 0.05 was considered significant.

## Results

### Patient characteristics

**Fig. 1:**
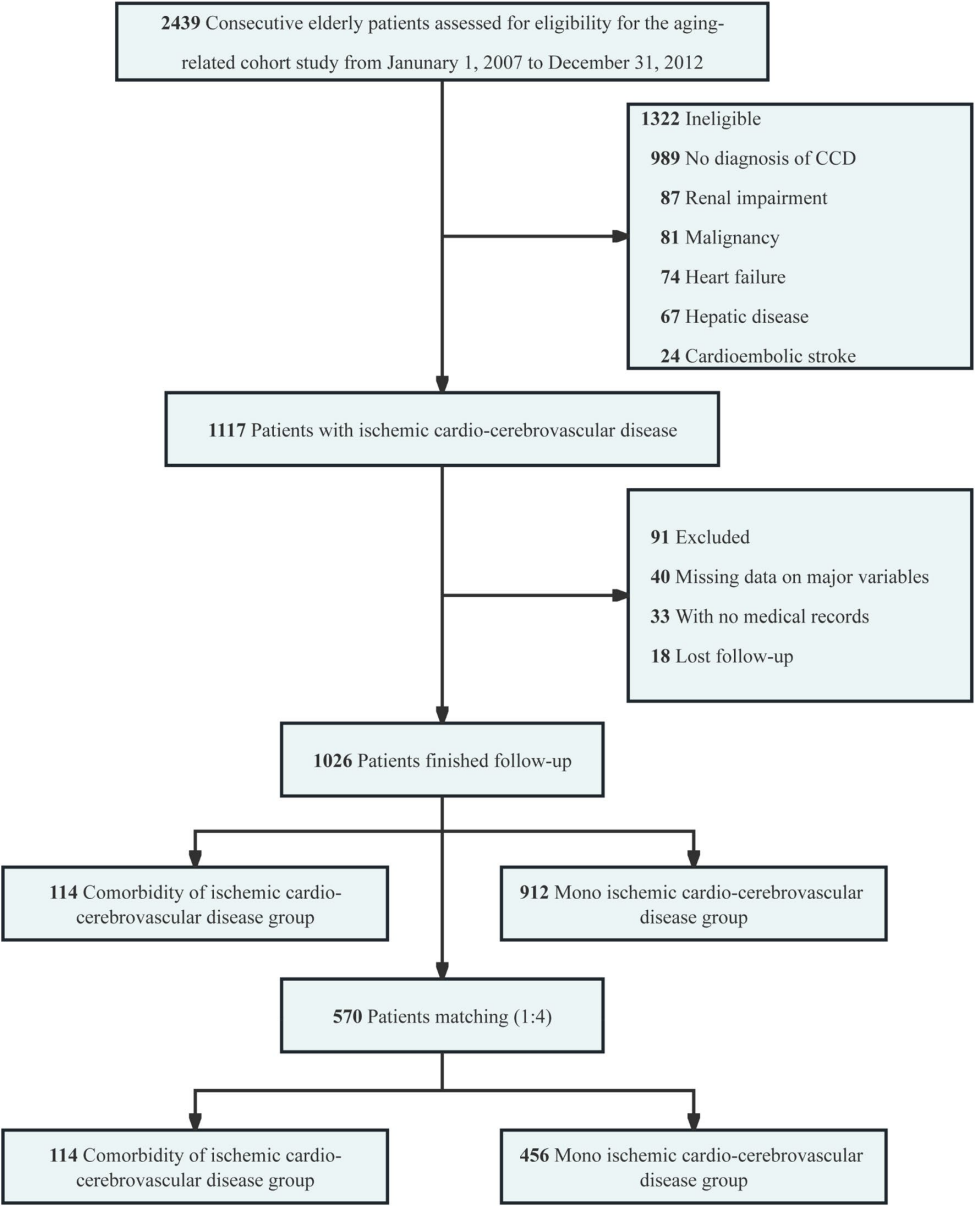
Eligibility of the study patients. The flowchart of the eligibility of 1026 patients is included in the long-term mortality analysis

Finally, as shown in Fig. 1, 1026 patients with ischemic cardio-cerebrovascular disease were included, of which 912 (88.89%) had MICCD and 114 (11.11%) had CICCD. The median (IQR) age at the baseline was 82.90 (76.53, 86.40) years, including 5.6% of women. A total of 889 individuals were diagnosed with coronary artery disease, and 251 were diagnosed with stroke or TIA. Table 1 shows the characteristics of the participants. Patients with CICCD were more likely to be older (84.65 [81.03, 87.65] vs. 82.65 [75.80, 86.32]; difference, 2.20 years; 95% CI 1.00–3.40 years; *P* < 0.001), had higher TC (4.22 [3.81, 4.93] vs. 4.06 [3.50, 4.71] mmol/L; difference, 0.20 mmol/L; 95% CI 0.03–0.36 mmol/L; *P* = 0.019) and HDL (1.25 [1.08, 1.47] vs. 1.17 [0.96, 1.41] mmol/L; difference, 0.07 mmol/L; 95% CI 0.01–0.14 mmol/L; *P* = 0.023). The CCI-score was higher in patients with CICCD (4.00 [3.00, 4.00] vs 3.00 [3.00, 4.00]; difference, 0.00; 95% CI 0.00–1.00; *P* < 0.001). Medication use on statins seemed to be more prevalent in CICCD patients (64.91% vs 50.77%; difference, 14.14%; 95% CI 4.80%–23.49%; *P* = 0.006).

Age, hemoglobin, CRP, creatinine, albumin, BNP, HDL, LDL, HbA1c, eGFR, and CCI-score differed significantly between patients without and with all-cause mortality, and the only exceptions were UA and TC (Supplementary Table 1). Smoking status, cancer, and use of ADP receptor inhibitors and CCBs were associated with all-cause mortality in univariable analysis (Supplementary Table 2). Nonlinear relationships between the selected continuous variables and the risk of all-cause mortality were tested using restricted cubic splines (for nonlinearity, *P* < 0.001 for BNP, *P* = 0.039 for eGFR).

### Variation in all-cause mortality

**Fig. 2:**
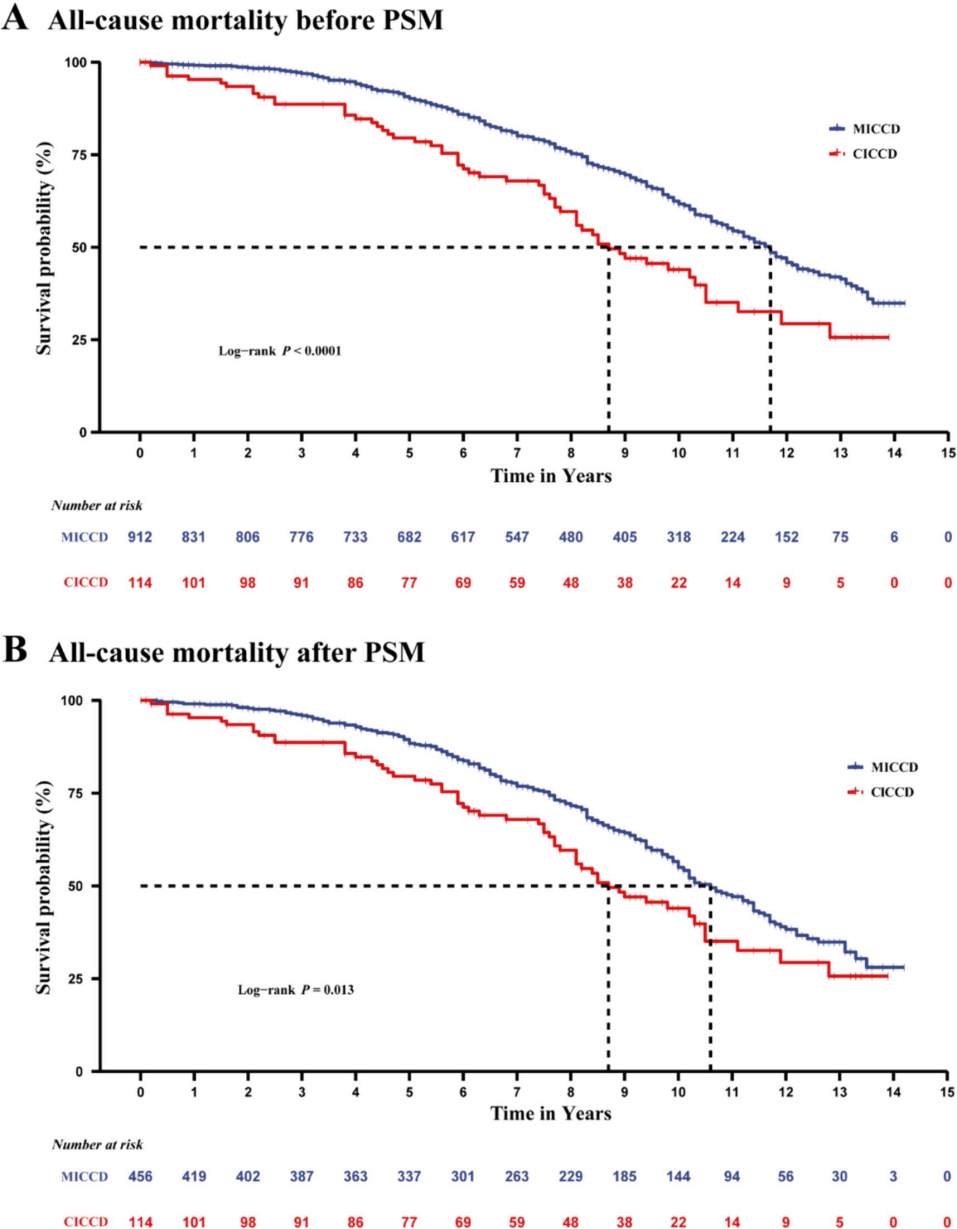
Kaplan–Meier curve for all-cause mortality between groups before and after PSM. **A** All-cause mortality before PSM. **B** All-cause mortality after PSM. MICCD, Mono ischemic cardio-cerebrovascular disease; CICCD, Comorbidity of ischemic cardio-cerebrovascular disease; PSM, Propensity-score matching

During a median follow-up of 10.4 (10.1–10.8) years, 398 patients experienced all-cause mortality. The incidence rate of all-cause mortality was 7.64 per 100 person-years among participants with CICCD and 4.84 per 100 person-years among participants with MICCD. Compared with patients having MICCD, those with CICCD had higher crude rates of all-cause mortality (Fig. 2A). After adjustment for age and other potential confounders (Table 2), CICCD was independently associated with a 71% increased risk of all-cause mortality (all-cause mortality: adjusted HR: 1.71; 95% CI 1.32–2.39). The results did not change in the sensitivity analysis (Table 3). In addition, anemia (adjusted HR: 1.48; 95% CI 1.16–1.89) and malnutrition (adjusted HR: 1.43; 95% CI 1.15–1.78) are also independent risk factors for all-cause mortality among the elderly and very elderly patients with ischemic cardio-cerebrovascular disease.

**Table 2:** Multivariable Cox regression for risk of all-cause mortality before and after PSM

Variables	Before PSM		After PSM	
	All-cause mortality	*P*-value	All-cause mortality	*P*-value
	HR 95%CI		HR 95%CI	
ICCD groups				
CICCD	1.71 (1.32–2.39)	** < 0.001**	2.07 (1.47–2.90)	** < 0.001**
MICCD	1 [Reference]		1 [Reference]	
Age	1.13 (1.11–1.16)	** < 0.001**	1.13 (1.09–1.18)	** < 0.001**
Gender				
Male	1.37 (0.85–2.20)	0.198	1.48 (0.79–2.76)	0.218
Female	1 [Reference]		1 [Reference]	
BMI				
Underweight	2.84 (1.70–4.73)	** < 0.001**	2.51 (1.21–5.20)	**0.013**
Overweight	0.91 (0.73–1.12)	0.369	0.85 (0.59–1.23)	0.386
Obese	0.69 (0.38–1.26)	0.227	0.32 (0.05–2.24)	0.253
Normal	1 [Reference]		1 [Reference]	
Smoking				
Current	1.51 (0.84–2.73)	0.167	1.89 (0.80–4.43)	0.146
Ever	1.30 (1.04–1.61)	**0.010**	1.00 (0.68–1.48)	0.995
Never	1 [Reference]		1 [Reference]	
Hemoglobin				
Anemia	1.48 (1.16–1.89)	**0.002**	1.68 (1.00–2.83)	0.051
Normal	1 [Reference]		1 [Reference]	
CRP	1.06 (1.01–1.11)	**0.018**	1.08 (0.99–1.18)	0.079
Albumin				
Malnutrition	1.43 (1.15–1.78)	**0.001**	1.61 (1.12–2.29)	**0.009**
Normal	1 [Reference]		1 [Reference]	
Triglycerides	1.17 (1.03–1.34)	**0.015**	1.35 (1.11–1.65)	**0.003**
HDL	0.70 (0.50–0.98)	**0.039**	0.79 (0.46–1.37)	0.823
LDL	1.10 (0.95–1.26)	0.212	0.97 (0.75–1.26)	0.823
HbA1c	0.98 (0.85–1.14)	0.799	0.95 (0.75–1.26)	0.654
BNP	1.00 (1.00–1.00)	** < 0.001**	1.00 (1.00–1.00)	0.091
eGFR				
< 60	1.40 (1.12–1.76)	**0.003**	1.14 (0.79–1.66)	0.488
≥ 60	1 [Reference]		1 [Reference]	
CCI score	0.89 (0.81–0.98)	**0.016**	0.95 (0.82–1.11)	0.523
Cancer				
Yes	1.63 (1.09–2.44)	**0.018**	9.19 (0.82–103.00)	0.073
No	1 [Reference]		1 [Reference]	
Hypertension				
Yes	0.98 (0.71–1.35)	0.910	1.17 (0.65–2.12)	0.597
No	1 [Reference]		1 [Reference]	
Aspirin				
Yes	0.90 (0.73–1.13)	0.369	1.06 (0.70–1.61)	0.783
No	1 [Reference]		1 [Reference]	
ADP receptor inhibitor				
Yes	1.00 (0.80–1.26)	0.971	1.11 (0.74–1.68)	0.604
No	1 [Reference]		1 [Reference]	
Statin				
Yes	1.02 (0.81–1.28)	0.891	0.92 (0.62–1.36)	0.659
No	1 [Reference]		1 [Reference]	
CCB				
Yes	1.38 (1.03–1.85)	**0.031**	1.10 (0.68–1.76)	0.708
No	1 [Reference]		1 [Reference]	

PSM, Propensity score matching; HR, Hazard ratio; CI, Confidence interval; BMI, Body mass index; CRP, C-reactive protein; BNP, B-type natriuretic peptide; HDL, High density lipoprotein; LDL, Low density lipoprotein; eGFR, estimated glomerular filtration rate; CCI, Charlson Comorbidity Index; CCB, Calcium channel blocker; ICCD, Ischemic cardio-cerebrovascular disease; MICCD, Mono ischemic cardio-cerebrovascular disease; CICCD, Comorbidity of ischemic cardio-cerebrovascular disease

**Table 3:** Risk of all-cause mortality according to ischemic cardio-cerebrovascular disease groups

	MICCD	CICCD
All-cause mortality, HR (95% CI)		
Cases, No.	340	58
Incidence rate, per 100-person-year	4.84	7.64
Model 1	1 [Reference]	1.58 (1.19–2.09)
Model 2	1 [Reference]	1.57 (1.19–2.08)
Model 3	1 [Reference]	1.71 (1.27–2.32)
Model 4	1 [Reference]	1.65 (1.21–2.25)
Model 5	1 [Reference]	1.74 (1.28–2.38)

MICCD, Mono ischemic cardio-cerebrovascular disease; CICCD, Comorbidity of ischemic cardio-cerebrovascular disease; HR, Hazard ratio; CI, Confidence interval. Model 1 was adjusted for age and gender. Model 2 was further adjusted for BMI and smoking. Model 3 was further adjusted for hemoglobin, CRP, albumin, BNP, triglycerides, HDL, LDL, Hba1c, eGFR, CCI, cancer, hypertension, medication use of aspirin, ADP receptor inhibitor, statin. Model 4 was sensitivity analysis using only male patients. Model 5 was sensitivity analysis excluding those who died within the 1st year of follow-up

### Ischemic cardio-cerebrovascular disease and all-cause mortality after propensity-score (PS) matching

Furthermore, after PS matching for prognostic factors that differed significantly between groups, a total of 114 cases from the CICCD group and 456 individuals from the control groups were considered for matched analysis. When the biases were eliminated by PS matching, the demographic and clinical characteristics were comparable between the two groups (*P* > 0.05; SMD < 0.10; Table 1). The median follow-up for the matched cohort was 10.1 years. In total, 334 patients experienced all-cause mortality. Compared with patients having MICCD, those with CICCD displayed higher rates of all-cause mortality (Fig. 2B). The fully adjusted model yielded similar results (all-cause mortality: adjusted HR: 2.07; 95% CI 1.47–2.90; Table 2).

### Subgroup analysis

**Fig. 3:**
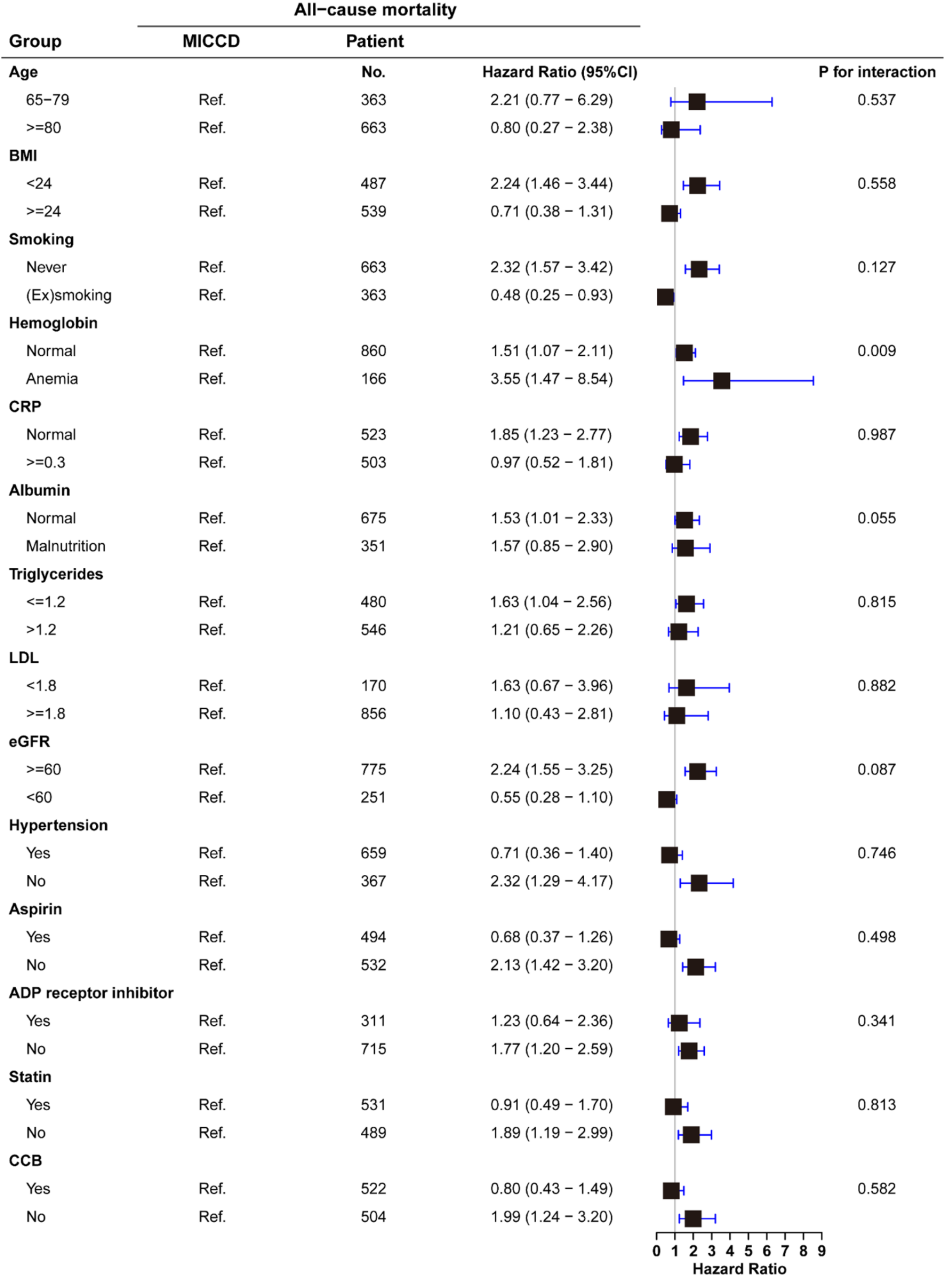
Subgroup analyses. Subgroup analyses for the adjusted HR (95% CI) of ischemic cardio-cerebrovascular disease groups for long-term mortality by age, BMI, smoking, hemoglobin, CRP, albumin, TG, LDL, eGFR, hypertension, aspirin, ADP receptor inhibitors, statins, and CCB. BMI, body mass index; CCB, calcium channel blocker; MICCD, Mono ischemic cardio-cerebrovascular disease; CI, confidence interval; CRP,  C-reactive protein; eGFR, estimated glomerular filtration rate; HR, hazard ratio; LDL, low-density lipoprotein; TG, triglycerides

In the subgroup analysis, significant interactions were not observed between CICCD groups and age, BMI, smoking, CRP, albumin, TG, LDL, eGFR, hypertension, medication use aspirin, ADP receptor inhibitors, statins, and CCBs with regard to all-cause mortality (*P*-interaction > 0.05 for all). The association of CICCD with all-cause mortality was modified by hemoglobin status (*P*-interaction = 0.009). An increased risk of CICCD with all-cause mortality showed among anemia status (HR: 3.55; 95% CI 1.47–8.54) (Fig. 3).

## Discussion

### Main interpretation

**Fig. 4:**
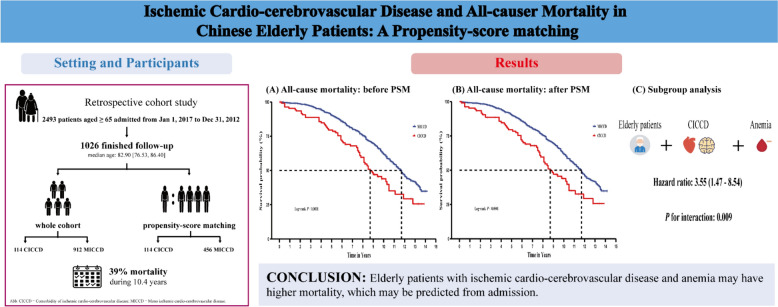
Graphical abstract. Left: A brief flowchart summary of this study. Right: Main results of this study. CICCD,  Comorbidity of ischemic cardio-cerebrovascular disease; MICCD,  Mono ischemic cardio-cerebrovascular disease

Cardiac/neurologic-related chronic diseases remain the main killer worldwide [23–29]. The results of this study provide valuable insights into the association between comorbidity of ischemic cardio-cerebrovascular disease and all-cause mortality among Chinese elderly patients (see Fig. 4). A significant increase was noted in the risk of all-cause death in patients with CICCD and anemia or malnutrition, which may be predicted from admission. These findings persisted after adjusting for various confounding factors and in sensitivity analyses. Our results agree with the impact of poly-vascular disease on mortality outcomes reported in several studies [30–32], further strengthening this evidence by specifically focusing on the Chinese elderly population and examining the influence of comorbidity of ischemic cardio-cerebrovascular disease.

Atherosclerosis is a complex systemic disease driven by intricate pathophysiological mechanisms affecting the vascular wall's intima [33]. With advancing age, the incidence of vascular diseases rises, drawing significant attention to the characteristics of poly-vascular disease. Despite this, there is a lack of long-term follow-up studies on elderly patients with comorbid ischemic cardio-cerebrovascular disease (CICCD). Comprehensive assessment of patients with atherosclerotic disease, including detailed medical history and thorough physical examination, is crucial. Recent studies [34] indicate that the incidence of poly-vascular disease increases with age, with approximately 3.0% of asymptomatic patients showing vascular lesions in 2–3 different sites, significantly elevating the risk of all-cause mortality. Our study corroborates these findings, revealing that patients with CICCD have a markedly higher risk of all-cause mortality, with differences in prognosis evident as early as the first year of follow-up and persisting long-term. Therefore, screening elderly patients for poly-vascular diseases is justified.

Despite the elevated all-cause death risk among individuals with CICCD, our results also identified that being underweight and elevated triglyceride are other significant factors. Elderly patients with ICCD and higher BMI have better outcomes than those with lower BMI, a phenomenon often referred to as the “obesity paradox” [35]. Triglyceride contributes to atherosclerosis development by promoting atherogenic lipoprotein formation and inducing endothelial dysfunction [36]. Subgroup analysis showed that CICCD only matters when triglyceride is controlled in an ideal condition, underscoring the importance of managing triglyceride levels in elderly patients with ischemic cardio-cerebrovascular disease to improve their long-term prognosis.

In the present study, we note that our clinical cohort included more than 90% male participants, and potential gender bias could affect the representativeness of the results. To minimize potential gender bias that could affect the representativeness of the results, we performed sensitivity and subgroup analysis for individual men and women. There were no substantial differences in the strength of association between coronary artery disease, ischemic stroke/TIA, or all-cause death risk factors and sex in elderly patients. Our results are similar to the historical reports [37, 38].

Anemia ranks as the fifth cardiovascular risk factor following smoking, diabetes, hypertension, and dyslipidemia [39]. The following may be the underlying mechanisms [40]: (1) reduction in tissue oxygen intake; (2) increased instability of the plaque; and (3) decreased nitric oxide secretion. Past studies have suggested anemia as an independent risk factor for adverse outcomes in elderly individuals [41, 42]. Notably, our investigation also identified an intriguing interaction between cardio-cerebrovascular comorbidities and anemia concerning all-cause mortality. Subgroup analysis alluded that anemia may exacerbate the impact of ischemic cardio-cerebrovascular comorbidities on mortality risk. Malnutrition is associated with poor prognosis in various illnesses. Previous studies have shown that malnutrition has a strong impact on both short-term and long-term mortality in older adults, especially those with coronary artery disease [43, 44]. Once it occurs, malnutrition can advance to a condition known as overt cardiac cachexia. This comprehensive wasting syndrome impacts all bodily systems, including muscles, fats, and bones [45]. The origins of cachexia are complex and may stem from inadequate nutrition. Research has proposed that systemic inflammation can lead to a generally catabolic state by increasing the breakdown of proteins and decreasing their synthesis [46, 47]. Additionally, inflammation might contribute to a loss of appetite. These combined outcomes can result in protein-energy malnutrition and a reduced body mass index. Identifying patients at risk for developing cachexia may be achieved through early screening for malnutrition and its characterization.

In this special population, the findings of this study have also identified anemia and malnutrition as independent risk factors for elderly patients diagnosed with ischemic cardio-cerebrovascular disease. The significant impact of these factors on prognosis suggests that they should be a focal point in clinical management. With population life expectancy increasing, our study provides new, detailed insights into the long-term prognosis of elderly and very elderly patients with ischemic cardiovascular and cerebrovascular disease. It will provide valuable guidance for clinicians to optimize the management and treatment of this vulnerable population.

### Limitations

The strengths of our study included the cohort design, confirmed medical reports, and long-term follow-up time. However, it should be noted that our study has several limitations. First, our investigation was conducted in a single medical center with over 90% male participants, and it lacked non-Asian elderly patients, thus limiting the generalizability of our findings to other ethnic or demographic groups. Second, all-cause mortality was used as the endpoint without distinguishing between cardiovascular and nonvascular deaths, which are inherently different in terms of their pathogenesis. Third, considering chronic kidney disease, diabetes, and peripheral artery disease are common comorbidities in elderly patients, we used the age-adjusted Charlson comorbidity index in the multivariate analysis to control confounders. Statistical analysis was not conducted on the degree of stenosis in peripheral vessels, renal arteries, and the abdominal aorta. Fourth, our study may include ischemic strokes from vascular dissection and moyamoya disease, potentially affecting the interpretation of the association between comorbidities of ischemic cardio-cerebrovascular disease and long-term outcomes in elderly patients, despite efforts to minimize non-atherosclerotic stroke impact.

### Conclusion

In conclusion, the presence of comorbidity of ischemic cardio-cerebrovascular disease in elderly patients is associated with a higher burden of comorbidities and a progressive increase in long-term adverse prognosis. Patients with CICCD suffering from anemia or malnutrition appear to have a greater risk of all-cause mortality. These findings emphasize the need to acknowledge the accumulating risk linked with poly-vascular disease in elderly patients, owing to its consequential impact on risk assessment and the allocation of intensive and preventive medical interventions.

### Supplementary Information

Additional file 1. Table S1. Comparison between participants with and without all-cause mortality for continuous variables.

Additional file 2. Table S2. Univariable Cox regression for the association of categorical variables with all-cause mortality.

## Data Availability

The data are on https://www.jianguoyun.com/p/DbltF1cQuaiFChjRlbkFIAA.

## Abbreviations

BMI: Body mass index
BNP: B-type natriuretic peptide
CCBs: Calcium channel blockers
CCI: Charlson Community Index
CICCD: Comorbidity of ischemic cardio-cerebrovascular disease
CI: Confidence interval
CKD: Chronic kidney disease
CRP: C-reactive protein
eGFR: Estimated glomerular filtration rate
HbA1c: Glycated hemoglobin
HDL: High-density lipoprotein
HR: Hazard ratio
IPTW: Inverse probability of treatment weights
IQR: Interquartile range
LDL: Low-density lipoprotein
MICCD: Mono ischemic cardio-cerebrovascular disease
PSM: Propensity-score matching
RAASi: Renin–angiotensin–aldosterone system inhibitors
TC: Total cholesterol
TG: Triglycerides
TIA: Transient ischemic attack
