# Supplementary material for: Ischemic cardio-cerebrovascular disease and all-cause mortality in Chinese elderly patients: a propensity-score matching study
Source: Eur J Med Res. 2024 Jun 15;29:330. doi: 10.1186/s40001-024-01929-x (PMC11179225; doi:10.1186/s40001-024-01929-x)
Supplement: Supplementary file 1 — Additional file 1. Table S1. Comparison between participants with and without all-cause mortality for continuous variables. [file 40001_2024_1929_MOESM1_ESM.docx]

**Supplementary Table 1.** **Comparison between participants with and without all-cause mortality for continuous variables.**

|  |  | **Mean Rank** | **Median** | **IQR** | **Z-score** | ***P*-value** |
| --- | --- | --- | --- | --- | --- | --- |
| Age |  |  |  |  |  |  |
| Censor |  | 414.18 | 82.90 | 76.53 – 86.40 | -13.486 | **< 0.001** |
| Death |  | 670.21 |  |  |  |  |
| Hemoglobin |  |  |  |  |  |  |
| Censor |  | 583.18 | 134.00 | 124.00 – 145.00 | -9.599 | **< 0.001** |
| Death |  | 401.98 |  |  |  |  |
| CRP |  |  |  |  |  |  |
| Censor |  | 474.10 | 0.30 | 0.13 – 0.57 | -5.351 | **< 0.001** |
| Death |  | 575.67 |  |  |  |  |
| Creatinine |  |  |  |  |  |  |
| Censor |  | 471.37 | 83.00 | 71.00 – 98.00 | -5.721 | **< 0.001** |
| Death |  | 579.97 |  |  |  |  |
| Urine acid |  |  |  |  |  |  |
| Censor |  | 505.30 | 340.00 | 283.02 – 397.00 | -1.114 | 0.265 |
| Death |  | 526.44 |  |  |  |  |
| Albumin |  |  |  |  |  |  |
| Censor |  | 582.29 | 41.70 | 38.62 – 44.50 | -9.341 | **< 0.001** |
| Death |  | 404.96 |  |  |  |  |
| BNP |  |  |  |  |  |  |
| Censor |  | 432.51 | 128.00 | 63.40 – 291.82 | -10.997 | **< 0.001** |
| Death |  | 641.29 |  |  |  |  |
| Total cholesterol |  |  |  |  |  |  |
| Censor |  | 521.58 | 4.08 | 3.55 – 4.75 | -1.097 | 0.273 |
| Death |  | 500.75 |  |  |  |  |
| Triglycerides |  |  |  |  |  |  |
| Censor |  | 501.05 | 1.24 | 0.91 – 1.65 | -1.690 | **0.091** |
| Death |  | 533.14 |  |  |  |  |
| HDL |  |  |  |  |  |  |
| Censor |  | 534.43 | 1.18 | 0.96 – 1.41 | -2.843 | **0.004** |
| Death |  | 480.47 |  |  |  |  |
| LDL |  |  |  |  |  |  |
| Censor |  | 531.25 | 2.42 | 1.97 – 2.99 | -2.410 | **0.016** |
| Death |  | 485.49 |  |  |  |  |
| HbA1c |  |  |  |  |  |  |
| Censor |  | 493.91 | 6.10 | 5.80 – 6.50 | -2.665 | **0.008** |
| Death |  | 544.41 |  |  |  |  |
| eGFR |  |  |  |  |  |  |
| Censor |  | 580.39 | 74.61 | 60.43 – 83.59 | -9.082 | **< 0.001** |
| Death |  | 407.96 |  |  |  |  |
| CCI score |  |  |  |  |  |  |
| Censor |  | 435.41 | 3.00 | 3.00 – 4.00 | -10.935 | **< 0.001** |
| Death |  | 636.72 |  |  |  |  |
