# Supplementary material for: Ischemic cardio-cerebrovascular disease and all-cause mortality in Chinese elderly patients: a propensity-score matching study
Source: Eur J Med Res. 2024 Jun 15;29:330. doi: 10.1186/s40001-024-01929-x (PMC11179225; doi:10.1186/s40001-024-01929-x)
Supplement: Supplementary file 2 — Additional file 2. Table S2. Univariable Cox regression for the association of categorical variables with all-cause mortality. [file 40001_2024_1929_MOESM2_ESM.docx]

**Supplementary Table 2.** **Univariable Cox regression for the association of categorical variables with all-cause mortality.**

|  | **All-cause mortality** |  |
| --- | --- | --- |
| **Variables** | **HR 95%CI** | ***P*-value** |
| Gender |  |  |
| Male | 1.25 (0.809 - 1.95) | 0.311 |
| Female | 1 [Reference] |  |
| BMI |  |  |
| Underweight | 2.23 (1.39 - 3.57) | **< 0.001** |
| Overweight | 0.89 (0.73 - 1.10) | 0.276 |
| Obese | 0.51 (0.28 - 0.91) | **0.023** |
| Normal | 1 [Reference] |  |
| Smoking |  |  |
| Current | 0.88 (0.50 - 1.54) | 0.656 |
| Ever | 1.32 (1.08 - 1.62) | **0.007** |
| Never | 1 [Reference] |  |
| Cancer |  |  |
| Yes | 1.58 (1.10 - 2.26) | **0.014** |
| No | 1 [Reference] |  |
| Hypertension |  |  |
| Yes | 0.89 (0.73 - 1.09) | 0.254 |
| No | 1 [Reference] |  |
| Atrial Fibrillation |  |  |
| Yes | 0.97 (0.66 - 1.43) | 0.886 |
| No | 1 [Reference] |  |
| Aspirin |  |  |
| Yes | 0.91 (0.74 - 1.11) | 0.337 |
| No | 1 [Reference] |  |
| ADP receptor inhibitor |  |  |
| Yes | 1.20 (0.98 - 1.48) | **0.082** |
| No | 1 [Reference] |  |
| Statin |  |  |
| Yes | 0.93 (0.76 - 1.14) | 0.494 |
| No | 1 [Reference] |  |
| Anticoagulant |  |  |
| Yes | 1.39 (0.66 - 2.93) | 0.390 |
| No | 1 [Reference] |  |
| Beta blocker |  |  |
| Yes | 1.15 (0.87 - 1.52) | 0.329 |
| No | 1 [Reference] |  |
| ACEI/ARB |  |  |
| Yes | 1.02 (0.83 - 1.24) | 0.885 |
| No | 1 [Reference] |  |
| CCB |  |  |
| Yes | 1.42 (1.16 - 1.73) | **< 0.001** |
| No | 1 [Reference] |  |
